# Stable Isotopes Unveil Dietary Trends in the Samnite and Peligni Communities of Opi Val Fondillo and Sulmona S. Lucia (V–VI Centuries BCE, Abruzzo, Central Italy)

**DOI:** 10.3390/biology12111382

**Published:** 2023-10-28

**Authors:** Iuri Icaro, Inmaculada Alemán, Joan Viciano

**Affiliations:** 1 Department of Legal Medicine, Toxicology and Physical Anthropology, University of Granada, Avenida de la Investigación 11, 18071 Granada, Spain; ialeman@ugr.es; 2 Independent Researcher in Physical Anthropology, Via Fiume 4, 65122 Pescara, Italy; joanviciano@gmail.com

**Keywords:** Iron age, pre-Roman period, italic population, bone collagen, stable isotopes, carbon, nitrogen, paleodiet

## Abstract

**Simple Summary:**

The study uses stable-isotope analysis to investigate the diets of ancient populations in Opi Val Fondillo and Sulmona S. Lucia, Italy, during the Vth and VIth centuries BCE. By examining stable isotopes of carbon and nitrogen in rib fragments, researchers can trace dietary habits, even in the last years of life before death. Ribs are representative of approximately the last five years of an individual’s life. This method helps in studying food habits, ecology, and cultural and environmental changes that occurred over time. Additionally, stable-isotope analysis is useful for studying diseases related to the nutritional or physiological conditions that individuals experienced.

**Abstract:**

The purpose of this study is to gain insights into the subsistence activities and nutrition of the Samnite and Peligni populations who lived in the Abruzzo region (Italy) during the Iron Age. The samples under investigation are from Opi Val Fondillo (AQ) and Sulmona S. Lucia (AQ), dating between the Vth and VIth centuries BCE. Carbon and nitrogen isotopes were utilized to characterize the diet of the inhabitants in this region. The study involved analyzing carbon and nitrogen stable isotopes in 84 available samples and comparing them with isotopic values from animals found in the nearby sites of Loreto Aprutino, Gabii, and La Sassa’s Cave. The results of this study revealed statistically significant differences between sexes in δ^15^N values. Additionally, significant statistical variations were observed when comparing different populations.

## 1. Introduction

Studying the diet preferences of ancient populations through stable-isotope analysis of carbon and nitrogen is a valuable method for understanding their food habits [1]. Stable carbon and nitrogen isotopes are particularly useful as they reveal information about the sources of dietary protein and the trophic levels within the food web [2]. Carbon isotopes, specifically ^13^C, can indicate the type of plants consumed, distinguishing between terrestrial (C_3_, C_4_) or marine resources with different values [2]. Nitrogen isotopes, specifically ^15^N, reflect an organism’s position within the food chain, with higher values indicating a higher trophic level [1]. In the Abruzzo region, situated in central-southern Italy, archaeological samples from the same time periods have been analyzed, and the isotopic signatures of the populations occupying that territory have been compared. By examining the carbon and nitrogen isotopes in human and faunal remains [1], researchers can reconstruct the dietary patterns and preferences of these ancient populations. The comparison involves selecting chronologically and geographically coherent populations from central-southern Italy, including the Abruzzo region. This means choosing populations that lived during similar time periods and occupied neighboring or overlapping geographical areas. By comparing the isotopic signatures of these populations, it becomes possible to identify differences and similarities in their diets. For instance, if one population shows higher carbon isotope values (indicating a reliance on C_4_ plants) compared to another population with lower values (indicating a predominantly C_3_ plant-based diet), it suggests differences in subsistence strategies or access to different food resources [2]. Similarly, variations in nitrogen isotope values can provide insights into variations in protein sources and trophic levels between the populations [1]. Through careful analysis and interpretation of stable-isotope data, researchers can reconstruct and compare the dietary preferences of past populations in the Abruzzo region and other central-southern Italian populations. This approach sheds light on the cultural, environmental, and economic factors that influenced food choices and subsistence strategies throughout history [1].

During the Iron Age in the Apennine Mountains, in a region of central-southern Italy called Samnium, a population known as the Samnites lived [3]. The territory was abundant in pastures and rivers but not suitable for agriculture due to its mountainous nature. While some traces of agriculture have been found, the primary economic activity was livestock breeding, specifically practiced through vertical transhumance. This involved moving cattle to higher mountains during the summer and down to lower areas in the winter. The livelihood of the Samnites appeared to be agro-pastoral [4]. It was a complex society built on multiple patrilinear alliances that served for family, livestock, and territorial protection. Epigenetic analysis with hereditary components suggests close relationships among people buried in the same funerary complex of these multiple-patrilineal alliances [4,5,6,7,8]. In the population of the Opi Val Fondillo necropolis, frequent pathologies indicated that male individuals engaged in dynamic and heavy work activities, while females had more sedentary work roles [9]. The Peligni, another ancient Italic people during the Iron Age, settled in the present-day region of Abruzzo, Italy [10]. Limited direct written sources exist about the Peligni, but archaeologists have reconstructed aspects of their society through analysis of archaeological findings, including tombs, settlements, and artifacts [11]. From a social perspective, the Peligni society seemed to be highly stratified, with a dominant aristocratic class and a larger population consisting of farmers and artisans [11,12]. Their society was organized into tribes, each led by a tribal chief who held political and military power. Monumental tombs, like those found in Alfedena and San Pelino, were likely reserved for members of the aristocratic elite and contained valuable funerary objects reflecting the prestige and wealth of the deceased [13]. Pelignian settlements featured regular urban planning with straight roads and orderly arranged houses typically built with stone and clay, having thatched or wooden roofs. Defensive fortification around the settlement indicated the need for protection and defense [11,12,13]. The Peligni economy primarily revolved around agriculture and livestock farming [11]. They cultivated crops such as wheat and barley and produced wine and olive oil [11,14]. The Peligni were skilled craftsmen, particularly in metalworking, with expertise in iron, bronze, and gold. The metal artifacts found in Pelignian tombs indicate a sophisticated artisanal tradition [11]. From a religious perspective, the Peligni practiced the cult of death and the nature deities [11,12]. Archaeological research on Pelignian sanctuaries, like Monte Pallano, suggests that they were sites for votive offering and rituals [11,12]. Overall, archaeological studies indicated that the Peligni society was relatively advanced, with a hierarchical structure consisting of a dominant aristocratic class, a solid peasant and artisan base, and agriculture, craftmanship, and religious worship as fundamental pillars of daily life [11,12,13,14].

Our understanding of the dietary habits of ancient populations primarily comes from: (i) literary sources; (ii) archaeological evidence such as faunal and plant remains, food preparation and conservation utensils, paintings, mosaics, and sculptures [1]; and (iii) indirect biological sources, like dental diseases [2]. While these sources provide some information about available foods, they do not provide specific details about the foods actually consumed. Therefore, stable-isotope analysis from human skeletal remains has become an increasingly popular technique in bioarchaeology [15], because it can identify the types of food individuals consumed and define their relative quantities in the diet. Stable-isotope analysis is highly relevant in reconstructing the evolution of dietary habits over time, considering factors such as age and environmental changes, as seen in populations during the Bronze and Iron Ages in central Italy. For example, sites like Gabii [16] and Loreto Aprutino [17], belonging to the Iron Age, and La Sassa Cave [18], dating back to the Bronze Age and the Copper Ages, are located in the same geographical area. The analysis of these sites using stable isotopes can provide valuable insights into the dietary practices of ancient populations during these periods in central Italy.

### 1.1. The Nutritional Investigation

The nutritional investigation is based on the principle that all ingested substances contribute to tissue formation, including bones, leaving traces that can be analyzed. The reconstruction of a dietary pattern can be achieved by examining skeletal and dental remains. Stable carbon and nitrogen isotopes in bone collagen are particularly useful for tracing the protein diet of ancient populations, as bone-isotope analysis can provide information about an individual’s diet in the last years of life [19]. The timespan covered by bone-isotope analysis varies depending on the specific skeletal area being examined, allowing the diet of an individual to be traced back to 5–15 years before death [20]. Stable-carbon-isotope (^13^C,^12^C) analysis is commonly used in reconstructing the eating patterns of ancient populations [21,22,23,24,25]. International reference standards are employed to determine the abundance ratios of stable isotopes, which may differ depending on the element being analyzed. These ratios are expressed as parts per mil (‰) using the delta notation (δ), relative to a reference material: atmospheric N_2_ (ambient inhalable reservoir, AIR) for nitrogen and the Vienna-PeeDee belemnite (VPDB) marine limestone for carbon [26,27]. Carbon isotopic values in plants are reflected in the tissues of consumers, allowing research to determine the sources of the protein component in their diet, such as plants from C_3_ and C_4_ photosynthetic pathways, by measuring the δ^13^C of the consumer’s bones [21]. The isotopic values of C_3_ and C_4_ plants are distinctly separate, making it possible to distinguish between the two categories with isotope analysis. The average δ^13^C value for current C_3_ plants is −26.5‰, while C_4_ plants have a value of −12.5‰ [28,29]. Plant consumer offset is around 5‰, for Δ^13^C, calculated according to Ferrio et al. (2005); values ranged from 17.5 to 21.6‰ for legumes and from 16.1 to 19.3‰ for cereals [30]. Nitrogen values in the organic component of the skeletal remains are closely related to diet since proteins are the only source of nitrogen. δ^15^N allows researchers to obtain information about the trophic level [31,32]. Controlled feeding experiments on omnivorous mammals have determined an offset of 4.8‰ for nitrogen-collagen isotope, with an uncertainty of 0.5‰, which remains constant for each step in the food chain [33]. In terrestrial environments, trophic chain steps are usually limited to 2 or 3, whereas in marine environments, there can be up to 3–5 trophic levels [34]. Marine fishes tend to show higher values for δ^13^C (−10/−15‰) and for δ^15^N (even >25‰) due to enrichment in heavy isotopes originating from marine carbonates [35,36]. However, distinguishing marine diets from terrestrial ones can be challenging, considering the values observed at the beginning of the marine food chain (e.g., sardines, garum) with δ^15^N values ranging from +7 and +18‰ [34]. Leguminous plants have a value around 0‰ for δ^15^N [37] as they are nitrogen-fixers of atmospheric N_2_, making them distinguishable among C_3_ plants. Isotope ratios can be influenced by metabolic and physiological alterations caused by diseases and a shortage of protein-rich foods [38].

### 1.2. The Archeological and Biocultural Context

#### 1.2.1. The Samnite Necropolis of Opi Val Fondillo

**Figure 1:**
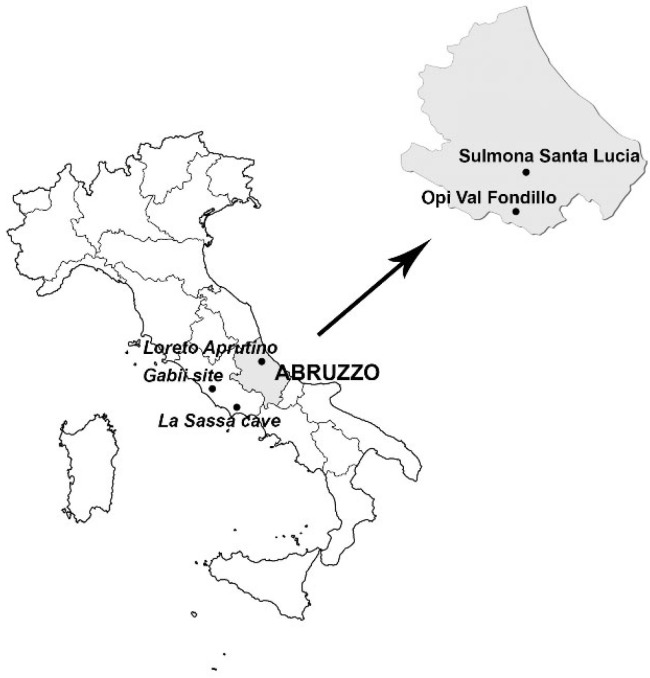
Geographical location of the necropolis of Opi Val Fondillo, Sulmona S. Lucia and Loreto Aprutino in the region of Abruzzo (Italy), and the Gabii and La Sassa cave sites.

Starting from the early Iron Age (around the IX century BCE), a population known as Safini inhabited the Abruzzo region and central Apennines. Over time, some clans from the *Safina* or *Sabina* populations split, leading to the formation of the *Marsi* clan [39]. The Safini population shares strong archaeological similarities with the entire Sangro valley, referred to as the “Sangritan culture” [40]. The Samnite necropolis of Opi Val Fondillo (41°46′44.4″ N 13°51′07.4″ E) (Figure 1) is situated at the confluence of the homonymous torrent and the Sangro river, where a widespread settlement based on an agricultural and forest-pastoral economy developed from the Archaic Age to the present day [41]. The upper Sangro valley served as the epicenter of this “culture”, with the Safini located at the center of trade between various peoples in central-southern Italy, thanks to a dense road network that exploited the territory’s morphology. The most significant and ancient North–South Road in Southern Italy traversed the Sangro valley, and, near Opi, another road branched off, leading directly to Lazio and crossing the Val Fondillo. Grave goods found in Opi Val Fondillo indicate interactions and contacts with people from neighboring territories, as evidenced by the presence of bucchero artifacts originating from the towns of Cales and Capua in the south and Lazio [42]. The necropolis of Opi Val Fondillo was used for funerary purposes from the VI to the V centuries BCE in a discontinuous manner. The burials can be classified into two types: box tombs and pit tombs. The box tombs consist of limestone slabs forming the sides and cover of the pit, which contains sand and gravel along with the deceased and their funerary equipment. The pit tombs, on the other hand, were dug into the gravel layer and then covered with earth [41]. The tombs were initially arranged in concentric circles, but from the V century onwards, they were arranged in rectangles at the borders of the necropolis, indicating changes in the political organization of the clans while maintaining ethnic and cultural continuity [40,42]. The buried bodies were placed supine, with arms and legs extended or crossed, and the head was typically oriented to the north or south, rarely to the east, and never to the west. The deceased were often wrapped in a shroud, leaving the head exposed and secured with fibulae along the body and leather laces at the feet [41]. The grave goods indicate significant variations in wealth, particularly in the personal funerary objects for both sexes. Female kits featured personal ornaments and specific objects related to the *mundus muliebre*, characteristic of the dignity of the role of women, such as the spindle whorl, likely used to balance the spinning wheel’s spindle during the spinning process, thus making the yarn more compact [41]. Other kits were commonly composed of impasto ceramics and iron fibulae. Men’s equipment, on the other hand, included offensive iron weapons and defensive bronze items, with the warrior’s panoply usually consisting of a sword and spear. Common offensive weapons were *gladii* with stamens or short daggers [41]. The study of settlements and funerary objects supports the presence of a stable population whose economy was linked to the forest-pastoral world [41]. At the moment, it is not possible to obtain archaeological information regarding the tombs due to the limited available material and encountered limitations. The only information relevant to our sample pertains to T36 sample 04, concerning the discovery of a large ironworking slag in the tomb’s fill. This may be an indication of the presence of ironworking activities among the populations in this part of the Val di Sangro [41].

#### 1.2.2. The Pelignan Necropolis of Sulmona S. Lucia

The territory inhabited by the Peligni people is located between the eastern mountain ranges of the central Apennines: the Gran Sasso–Monte Morrone–Majella group and the group extending from Monte Terminillo, passing through Monte Sirente, to La Meta [10]. The natural center of the region is the Sulmona basin, also known as the Conca Peligna. This basin, the lowest among all the basins in the Abruzzo region, takes the form of a triangular basin surrounded by limestone massifs [11]. During the Villafranchian period, it was occupied by a lake that later drained through the Gola di Popoli, leaving behind rich deposits of sand, clay, and pebbles at the basin’s bottom. These deposits, subsequently covered by cemented debris, allowed the Peligni people to dig tombs in the area [11].

The necropolis of Sulmona S. Lucia (Fonte d’Amore) (42°04′53.5″ N 13°55′42.3″ E) is located in the Peligna area and dates back to the IV century BCE. It is situated in the foothills of Monte Morrone, historically serving as a natural boundary between the Peligni people, who inhabited the area, and the Marrucini. The necropolis indicates a long-lasting presence and occupation of the area, spanning from the Protohistoric period to the late Imperial era. The discovery of the Santa Lucia necropolis, slightly southeast of the Fonte d’Amore village, in a breccia quarry, confirms the existence of a large settlement in the area. Numerous “grotticella” tombs were found in this necropolis [11], which were characteristic of the Peligni region and common in various parts of Abruzzo during the Hellenistic period [43]. The distribution of the burials suggests the presence of family groups, without strict internal regulation of burial spaces, adapting to the rocky terrain’s nature. The tomb types and funerary offerings show progressive assimilation to forms and rituals common among the central Italian populations, while still reflecting respect for local traditions and cultural identity. The grave goods are often modest and standardized, mainly consisting of various types of jewelry and bronze artifacts, iron weapons, and ceramic vessels of varying refinement [44]. The overall picture about archaeological information suggests a society with moderate-to-poor economic conditions, with some social stratification evident in personal belongings and gender-specific items [44]. Burials of children also follow customary associations with Peligni grave goods but on a smaller scale. The “grotticella” tombs in Fonte d’Amore date from the late IV century BCE to the I century CE [44]. A group of burials from the late III to the first half of the II century BCE exhibits significant innovations compared to earlier phases, both in terms of grave goods and tomb typology. Burials from the late II to the first half of the I century BCE show further variation due to the presence of imported items, such as amber objects. From this period onward, the area was likely incorporated into the sphere of influence of the Sulmo municipium. Between the last Ist century BCE and the Ist century CE the cemetery area expanded towards the north, reflecting the growing cultic and sacred significance of the region, closely connected to the monumentalization of the nearby sanctuary of Hercules [45]. The analysis of the necropolis and funerary offerings indicates a community based on subsistence economy closely tied to agricultural and pastoral activities [11]. It is plausible to think that this necropolis was associated with a pagus (a rural district) rather than the ancient city of Sulmo, considering the distance between the Fonte d’Amore area and the city. The expansion of the burial area in later chronological phases corresponds to the dynamics of settlement and reflects the growing cultic and sacred significance of the region, associated with the nearby sanctuary of Hercules [45].

The objectives of this research are to characterize the diet of a human sample selected from Iron Age populations of Opi Val Fondillo and Sulmona S. Lucia. The study aims to explore variations in diet concerning the sex and age of the individuals within these populations. Additionally, the research seeks to compare the isotopic evidence of the dietary patterns with those of other populations (Loreto Aprutino, Gabii, and La Sassa cave) belonging to a different chronological period but geographically close. The isotopic data obtained from the analysis will be combined with paleopathological and archaeological data to gain insights into the lifestyle and dietary habits of the populations under investigation.

## 2. Materials and Methods

### 2.1. Samples

The skeletal samples for this study were obtained from two different necropolises in central-southern Italy. The Opi Val Fondillo sample comprises 48 individuals from the circle tombs within the Opi Val Fondillo necropolis. The Sulmona S. Lucia sample consists of 36 adult individuals from the pit tombs inside the Fonte D’Amore necropolis.

For both samples, sex estimation was conducted using the pelvis and cranial morphological characteristic methods [46], along with the population-specific odontometric method of Viciano et al. [47]. Age at death was determined by combining the methods of Brothwell [48] and Lovejoy et al. [49]. The wear of the occlusal surface of teeth was taken into consideration, as it progressively increases with age. Table 1 presents the age distribution of the examined sample. The individuals were categorized into three age groups following the conventional anthropological categories [50]: young adults (from 20 to 35 years of age); middle adults (from 35 to 50 years of age); and old adults (50 years of age and older).

### 2.2. Analytical Methods

The stable carbon and nitrogen isotope analysis was performed on 84 human bone samples. Rib fragments, approximately 1g each, were collected from each individual. The bone surface was cleaned using a sterile surgical blade, and the sample was pulverized with a drill. The protein fraction was extracted following the modified Longin protocol [51]. About 0.5 g of powdered bone were demineralized in 0.6 M HCl for 2 days at 4 °C. Afterward, the acid was removed using ddH_2_O and the remaining residue was gelatinized with 0.001 M HCl at 65 °C for 24 h. The resulting solute was frozen at −80 °C for 4 h and then freeze-dried for 1 to 2 days. Modern bovine bone collagen was used as a reference control, and each extraction run was performed simultaneously on the reference material. For each extract, 0.8–1.2 mg of collagen was weighed, placed into tin capsules, and analyzed in duplicate for δ^13^C and δ^15^N using an elemental analyzer isotope ratio mass spectrometer (EA-IRMS) at the IGAG laboratory CNR Roma–Monte Libretti. The analytical precision for δ^15^N was ±0.3‰ relative to the AIR, and ±0.1‰ for δ^13^C relative to the VPDB standard. The quality of preservation of the samples was assessed based on carbon content (C%), nitrogen content (N%), and C/N ratio [19,49,50], following criteria proposed by DeNiro [52] and van Klinken [53]. The samples were processed at the BiGeA Laboratory of the University of Bologna.

### 2.3. Statistical Analysis

The data were subjected to statistical analysis using IBM SPSS Statistics 25.0 software for Windows [54]. Normality of the data and homogeneity of variance were tested using Kolmogorov–Smirnov one-sample tests, and Levene tests, respectively, with a significance level of *p* ≤ 0.05. These tests were necessary to ensure the assumptions required for later tests.

Differences in the mean values of δ^13^C and δ^15^N between males and females in both skeletal samples, Opi Val Fondillo and Sulmona S. Lucia, were analyzed using the independent Student’s *t*-test and Mann–Whitney *U*-test. The appropriate test was chosen based on the fulfilment of assumptions. Additionally, the main effects of age groups on stable isotopes were tested using the non-parametric one-way Kruskal–Wallis *H* analysis.

Furthermore, the mean values of δ^13^C and δ^15^N for the Opi Val Fondillo and Sulmona S. Lucia samples were compared with those of Loreto Aprutino, Gabii, and La Sassa cave sites using the nonparametric Mann–Whitney *U*-test.

## 3. Results

### 3.1. Bone Collagen Analysis

**Figure 2:**
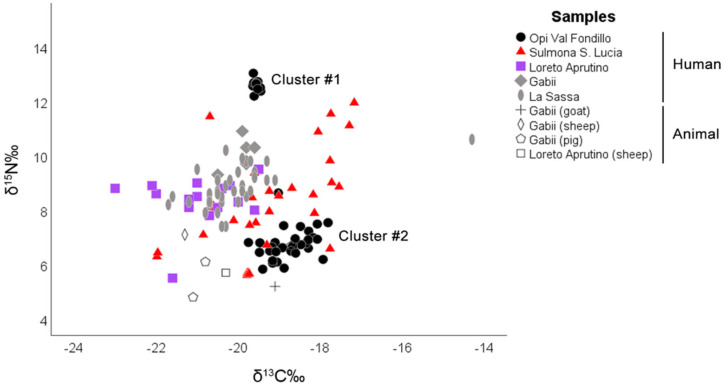
The plots of δ^13^C versus δ^15^N values for the studied human and animal specimens.

The stable-isotope data for the human specimen are presented in Table 2. A collagen yields higher than 1% was obtained for 72 of the 84 analyzed samples, indicating satisfactory protein quality indicators [52,53]. Figure 2 shows the plot of δ^13^C versus δ^15^N values for the studied human specimens. Since faunal remains were absent in the investigated area, isotopic data from animal samples from the site of Gabii [16] and Loreto Aprutino [17], belonging to the same chronological period, and La Sassa cave [18] from the same geographical area as our skeletal sample, were used for comparison. Table 3. shows the faunal values considered for the study. The choice was made to consider the three sites for comparison based on ecological and environmental similarities that would be reflected in the isotopic values of the animals considered for comparison. This decision was made because there were no animal remains associated with the necropolises under analysis, and also due to data availability. The δ^13^C values for individuals from Opi Val Fondillo ranged from −19.7‰ to −17.8‰ (median −19.03‰ Q1: −19.5‰, Q3: −18.5‰), while the δ^15^N values ranged from 6‰ to 12.7‰ (median 6.8‰, Q1: 6.5‰, Q3: 8.3‰). For individuals from Sulmona S. Lucia, the δ^13^C values ranged from −21.9‰ to −17.1‰ (median: −19.57‰, Q1: −18.1‰, Q3: −19.8‰), and the δ^15^N values ranged from 5.6‰ to 11.9‰ (median: 8.02‰, Q1: 6.8‰, Q3: 8.8‰).

In the Opi Val Fondillo sample, it is interesting to note the presence of two very different clusters in Figure 2. Cluster #1 included 10 individuals (sample ID Nos. O4, O5, O6, O13, O15, O18, O19, O21, O41, and O42) with high levels of nitrogen compared to the rest of the individuals in cluster #2. Cluster #1 showed low dispersion in δ^13^C and δ^15^N values, while cluster #2 included individuals with higher dispersion for these isotopic values, forming two distinct clusters. Both clusters were heterogenous, including individuals of both sexes and different age groups. The comparison between cluster #1 vs. cluster #2 revealed statistically significant differences for δ^13^C (Mann–Whitney *U*-test = 14.00; *p* = 0.000) and δ^15^N values (Mann–Whitney *U*-test = 0.00; *p* = 0.000).

### 3.2. Differences between Sexes

The Kolmogorov–Smirnov test showed that δ^13^C values were normally distributed (*p* > 0.05), while δ^15^N values were not (*p* < 0.001). The homogeneity of variance test results indicated that the sample was statistically homogeneous for both δ^13^C and δ^15^N values (*p* > 0.05). Table 4 presents the sample size, mean and standard deviation, *t*-value, *U*-value, and the significance level of the differences between male and female individual means for δ^13^C and δ^15^N data. For the Opi Val Fondillo sample, the results for δ^13^C values showed a higher value in males compared to females, but this difference was not statistically significant. However, for δ^15^N values, males showed significantly higher values compared to females (*p* < 0.05). As for the Sulmona S. Lucia sample, there were no significant differences observed in terms of carbon and nitrogen related to sexes.

### 3.3. Differences between Age Groups

Because some assumptions were violated (e.g., non-normal distribution for δ^15^N values, and unbalanced sample sizes for both stable isotopes within the age groups) the use of the one-way ANOVA was unappropriated. Thus, the non-parametric one-way Kruskal–Wallis *H*-test was applied. Results of the Kruskal–Wallis *H* analysis revealed no statistically significant differences between different age groups for δ^13^C and δ^15^N values (*p* > 0.05) (Table 5).

### 3.4. Differences between Opi Val Fondillo and Sulmona S. Lucia

The applied statistical Mann–Whitney *U*-test shows in the Table 6. non-significant differences regarding nitrogen and carbon between the two compared populations. 

### 3.5. Comparison between Opi Val Fondillo and Sulmona S. Lucia with Loreto Aprutiono, Gabii, and La Sassa Cave Sites

Table 7 presents the sample size, mean, standard deviation, *U*-value, and significance level of the differences between the mean values of δ^13^C and δ^15^N for the comparison of Opi Val Fondillo and Sulmona S. Lucia vs. Loreto Aprutino, Gabii, and La Sassa cave sites. For δ^13^C values, the results showed that Opi Val Fondillo had higher values compared to Gabii and La Sassa, and these differences were statistically significant (*p* < 0.05). However, there were no statistically significant differences between Opi Val Fondillo and Loreto Aprutino (*p* > 0.05). Sulmona S. Lucia had higher δ^13^C values compared to Loreto Aprutino and La Sassa, and these differences were statistically significant (*p* < 0.05). However, there were no statistically significant differences between Sulmona S. Lucia and Gabii (*p* > 0.05). For δ^15^N values, Opi Val Fondillo had lower values compared to Gabii, Loreto Aprutino, and La Sassa, and these differences were statistically significant (*p* < 0.05). Sulmona S. Lucia also had lower δ^15^N values compared to Gabii and La Sassa, and these differences were statistically significant (*p* < 0.05). However, there were no statistically significant differences between Sulmona S. Lucia and Loreto Aprutino (*p* > 0.05). In summary, the comparison of isotopic values between Opi Val Fondillo and Sulmona S. Lucia with the other sites (Gabii, La Sassa, and Loreto Aprutino) showed significant differences in δ^13^C and δ^15^N values, indicating variations in the dietary patterns and subsistence strategies among these populations. For Sulmona S. Lucia, higher deviations in isotopic values (higher SD) are observed than in the first necropolis if the two clusters are taken separately.

## 4. Discussion

Table 8 shows the median values of stable isotopes for the entire population considered in this study. The overall range of isotopes in the Opi Val Fondillo community is consistent with a diet primarily relying on a low intake of animal proteins and a mix of C3 and C4 resources. However, there were individuals within the sample, belonging to cluster #1, who showed higher nitrogen values, possibly indicating a higher intake of animal proteins or a low consumption of legumes. The diet of this population was likely based on terrestrial resources such as legumes and graminoids [14], along with occasional animal protein consumption. The analysis also showed no significant differences in isotopic values based on age groups, but there were significant differences in δ^15^N values between males and females. Males had higher δ^15^N values, possibly suggesting a higher consumption of animal proteins or specific health and physiological factors. The consumption of C_4_ plants could not be excluded for individuals showing δ^13^C values higher than −18‰. As suggested by previously published works [55,56], although debated in terms of importance [47], the presence of C_4_ plants should be taken into consideration. Literary sources refer to the use of millet, a C_4_ plant, as animal fodder, but it was considered less desirable for human nutrition under normal conditions of nutrient availability [56]. The low-nitrogen values recorded for the majority of the sample, but not for individuals belonging to cluster #1, might be indicative of a small intake of animal proteins and/or a high consumption of legumes [57] as the values under examination suggest. We would expect a diet based mainly on proteins of terrestrial origin; in fact, in cluster #2, the low nitrogen value could come from an almost usual consumption of *Sus scrofa* in the diet. The animals consumed belonged to the farmed livestock, or they could have been obtained through hunting or fishing activities. It is possible to hypothesize, given the geology and hydrography of the area, the possible consumption of anadromous fish, such as *Anguilla anguilla* (−11.3 ± 2.7; 8.4 ± 1.4) [58] or fish of the genus *Oncorhynchus* (−18.2 ± 1.4; 11.1 ± 2.1) [35], which have more positive carbon values, given their marine origin. Alternatively, due to their location along important trade routes, their diet could have been influenced by exotic foods, including those of marine origin. In this sense, in the Opi Val Fondillo necropolis, it is interesting to highlight the presence of a highly differentiated cluster (cluster #1) that is separated from the rest of the individuals. A relationship can be hypothesized between the individuals that make up this cluster, and for this reason their diet is similar with little variation between them, in accordance with their δ^13^C and δ^15^N values. However, due to the inability of the authors to access archaeological information (e.g., related to the distribution of the burials in the necropolis, and information on grave goods), it was not possible to infer whether this clear separation was due to a close kinship relationship of the individuals (it could be hypothesized that the same family nucleus had access to the same resources, other isotopes, such as oxygen and strontium, could also shed light on the origins of the people of cluster #1) or for sociocultural reasons. To determine if there was a family link between the samples under study, it would be advisable to deepen the analyses through the study of ancient DNA. To determine if there were social or cultural reasons, full access to archaeological information is required.

The overall isotope range of the Sulmona S. Lucia community is compatible with an omnivorous diet; the consumption of C_4_ plants cannot be excluded for individuals showing δ^13^C values higher than −18‰. The samples under examination exhibit a high variability in terms of δ^13^C. Regarding δ^15^N, the values range between +6‰ and +12.7‰. This could indicate differential access to resources, but no significant differences are found for δ^15^N, neither in terms of age groups nor regarding sex differentiation. Along with archaeological remains, this information can help define the social structure of the buried individuals in Sulmona S. Lucia [11]. In Sulmona S. Lucia, which belongs to a population with known social stratification [43,44,45], higher deviations in isotopic values (higher SD) are observed than in the first necropolis if the two clusters are taken separately. This could likely be interpreted by considering different factors of variability that may reflect the complexity of dietary and metabolic dynamics within the population. Taking into account the variability in diet, geographic variations, and individual differences, samples S7, S14, and S15 show high levels of nitrogen and low levels of carbon compared to the population average. This could suggest several possible interpretations: a diet with a strong component of marine proteins; a specialized diet; or a specific food source associated with low carbon values. This may be attributed to factors such as a diet based on C_3_ plants, which tend to have lower carbon isotope values compared to C_4_ plants. On the other hand, samples S1 and S5 display low nitrogen levels while having higher carbon levels compared to the population average. This could be attributed to a diet based on C4 plants, arid terrain, or, more specifically, a vegetarian diet. The high social stratification derived from archaeological information regarding the necropolis of Sulmona S. Lucia allows us to assess the variability within the population. It would be interesting to relate the isotopic analyses to the context of each individual burial and the funerary goods to provide a more accurate interpretation of the data. Unfortunately, due to the unavailability of specific tomb-related data at this time, we can only hypothesize and not precisely delineate the correlation between social status and diet. The earliest findings that indicate continuity in the studied area belong to the late Apennine Bronze Age, specifically the Sub-Apennine and Protovillanovan phases [11]. Decorative pottery motifs suggest that a significant number of discoveries can be attributed to the Sub-Apennine style [11]. Ceramic fragments found on the surface can be dated to both the Iron Age and the Bronze Age [11]. A more systematic examination of the Sulmona S. Lucia site (Fonte d’Amore) indicates its placement during the transition to the Iron Age. Contrary to general trends in Italy, most of the remains from the Sub-Apennine and Protovillanovan phases in Abruzzo are found in areas unsuitable for agriculture [11,12,13]. This is particularly evident in the Peligno territory, where such remains are located along the coast of Mount Morrone, on plateaus, and along access routes to the plateaus [11]. It is worth considering whether the influence of Sub-Apennine and Protovillanovan elements in Abruzzo resulted in a predominantly agricultural economy. The majority of findings are situated along major transit routes, suggesting a nomadic nature of the practiced livestock farming [11,12,13]. Comparing the population of Opi Val Fondillo with that of Sulmona S. Lucia, considering the breakdown by age groups and sex, no statistically significant differences are observed. Therefore, given the proximity of the two populations under study and their shared environment, we can hypothesize that the diet was very similar between the two populations, and was primarily based on agriculture and livestock farming.

Among the sites considered for comparison with our samples, we have the ancient Gabii site (V–VI BCE; [16]), the La Sassa Cave site (V–VI BCE; [18]), and the Loreto Aprutino site (IV–VI BCE; [17]. Gabii belongs to the same chronological period as our sample, while the La Sassa cave is dated between the Copper Age and the Late Bronze Age. Both are located in the Lazio region, near the territory from which the sample under examination originate. Loreto Aprutino belongs to the same chronological period as the populations under study, sharing the same territory as the Vestini, an Italic population that allied with the Peligni in the IV BCE to resist Rome. From the statistical analyses we conducted, it is evident that there are statistically significant differences between the samples under examination and the three sites compared. In the Gabii site, there was a higher access to animal proteins, indicated by the higher nitrogen values and lower carbon values. For the La Sassa cave, nitrogen values are very similar to the sample under examination while carbon values are lower. Access to resources is likely also influenced by the territory from which these populations originated. Further analyses will confirm and deepen our understanding of this topic. Sulmona S. Lucia exhibits differences in terms of carbon compared to Loreto Aprutino, while nitrogen values are similar. The information obtained from this study will be contributed to a broader project aimed at reconstructing the subsistence, cultural, and socio-economic activities of the populations that inhabited Abruzzo during the archaic era.

## 5. Conclusions

This study, conducted through the analysis of stable nitrogen and carbon isotopes, aimed to reconstruct the dietary patterns of the Opi Val Fondillo and Sulmona S. Lucia (Fonte d’Amore) communities in Abruzzo, Central Italy. The isotopic results suggest that their diet primarily depended on terrestrial resources, including both C_3_ plants and animal products. The use of C_4_ plants cannot be ruled out, as indicated by the carbon values. Within the Opi Val Fondillo sample, there is a significant difference in the δ^15^N levels observed in cluster #2, in which male individuals exhibit higher levels compared to females, a difference that holds when considering the entire population. Cluster #1, however, does not display any significant difference in terms of δ^13^C and δ^15^N. In summary, the data could imply a relatively uniform access to the resources among the studied population, while individuals within cluster #2 may have had distinct access to available resources. Regarding Sulmona Santa Lucia necropolis, on the other hand, in relation to the high social stratification of society based on the discovered burial assemblages, there were also findings of amber objects. Amber, an exotic material, could have been transported along the main trade routes from the coast into the inland area and along the routes connecting with Rome. It should be considered, given the isotopic values of certain individuals buried in Sulmona Santa Lucia, as reported in the discussion, that hypotheses related to marine-derived foods, possibly transported along the major trade routes, may be substantiated. Given the hydrography of the area, it would be worthwhile to investigate the relationship between the population and rivers and lakes to determine if these water sources played a significant role in providing food resources and if they were indeed exploited, or investigate, given the values compatible with seafood consumption from saltwater sources, whether there was consumption of such food coming from the Adriatic emporiums that then reached Opi and Sulmona through the Val di Sangro. This information will prove valuable for a broader project aimed at reconstructing the dietary, social, and economic behaviors of the inhabitants of Abruzzo during the Archaic era.

## Figures and Tables

**Table 1:** Distribution of the original and final sample by sex and age group from Opi Val Fondillo and Sulmona S. Lucia sites.

				Age Groups				
Sample		Sex		20–35 Years	35–50 Years	>50 Years	Adult	TOTAL
Opi Val Fondillo	(a)	Original sample						
			Male	16	9	1	0	26
			Female	7	9	5	1	22
			TOTAL	23	18	6	1	48
	(b)	Final sample						
			Male	15	7	1	0	23
			Female	7	7	5	0	19
			TOTAL	22	14	6	0	42
Sulmona S. Lucia	(a)	Original sample						
			Male	5	6	6	4	21
			Female	4	5	3	3	15
			TOTAL	9	11	9	7	36
	(b)	Final sample						
			Male	5	6	5	1	17
			Female	3	5	3	2	13
			TOTAL	8	11	8	3	30

**Table 2:** Isotope values (‰) of human samples (rib fragments) from Opi Val Fondillo and Sulmona S. Lucia sites.

Sample	ID No	Sex	Age (Years)	%C	%N	δ^15^N (‰)	δ^13^C (‰)	C/N Ratio	% Collagen Yield
Opi Val Fondillo	O1	F	55–60	39.7	14.3	6.81	−19.10	3.2	1.8
	O2	F	>45	— ^a^	—	—	—	—	—
	O3	F	50–55	42.8	15.6	6.09	−19.05	3.2	1.9
	O4	F	20–25	42.1	15.0	12.72	−19.52	3.3	2.0
	O5	F	50–55	40.4	14.3	12.65	−19.56	3.3	1.7
	O6	F	>45	41.9	15.0	12.45	−19.52	3.3	1.6
	O7	F	AD ^b^	—	—	—	—	—	—
	O8	F	35–39	—	—	—	—	—	—
	O9	M	25–26	38.5	13.8	6.95	−18.61	3.3	1.7
	O10	F	>50	24.5	8.2	6.62	−18.92	3.4	1.5
	O11	M	45–50	43.9	15.9	6.64	−19.18	3.2	1.8
	O12	F	35–40	42.5	15.6	6.60	−18.29	3.2	1.8
	O13	F	25–30	42.4	15.0	12.37	−19.45	3.3	2.1
	O14	F	40–45	43.2	15.7	6.78	−18.35	3.2	1.8
	O15	M	22–24	42.5	15.0	12.56	−19.64	3.3	1.3
	O16	M	>45	41.7	14.7	7.23	−18.28	3.3	1.3
	O17	M	35–39	—	—	—	—	—	—
	O18	M	22–24	41.4	14.5	13.03	−19.63	3.3	1.6
	O19	M	35–39	41.2	14.8	12.19	−19.61	3.3	1.4
	O20	F	>50	43.4	15.8	7.43	−18.89	3.2	1.5
	O21	M	45–50	42.1	15.0	12.68	−19.62	3.3	1.3
	O22	M	27–30	43.2	15.6	6.80	−19.47	3.2	1.4
	O23	M	>50	43.2	15.3	6.63	−18.62	3.3	1.6
	O24	M	27–30	32.8	11.7	6.99	−18.17	3.2	1.6
	O25	M	20–21	17.6	6.3	7.49	−18.07	3.2	1.5
	O26	F	35–39	43.9	16.1	6.50	−19.23	3.2	1.7
	O27	M	25–26	32.9	12.0	6.91	−18.29	3.2	1.8
	O28	M	30–35	43.4	15.5	7.54	−17.81	3.3	1.6
	O29	M	25–26	—	—	—	—	—	—
	O30	F	35–39	42.7	15.4	6.05	−19.16	3.3	1.7
	O31	M	>45	—	—	—	—	—	—
	O32	F	>45	29.6	9.5	6.73	−18.58	3.6	2.0
	O33	F	27–30	30.3	9.9	5.83	−19.40	3.6	1.7
	O34	F	18–25	43.9	16.1	5.87	−18.88	3.2	2.0
	O35	M	25–35	41.5	15.2	6.45	−19.48	3.2	2
	O36	M	25–35	41.1	14.6	6.47	−19.08	3.3	2.3
	O37	M	45–50	44.6	16.3	6.93	−18.07	3.2	1.7
	O38	M	25–26	35.5	12.9	6.73	−18.47	3.2	1.8
	O39	M	25–35	32.0	11.7	6.68	−18.67	3.1	2.1
	O40	M	39–44	42.3	15.0	7.40	−18.48	3.3	1.7
	O41	M	25–26	40.6	14.5	12.66	−19.56	3.3	1.7
	O42	M	45	40.6	14.3	12.50	−19.46	3.3	2.3
	O43	M	25–35	40.4	14.4	6.49	−18.71	3.3	1.6
	O44	F	39–44	42.6	15.4	6.19	−17.93	3.2	1.8
	O45	M	25–26	43.5	15.7	8.63	−19.02	3.2	1.8
	O46	F	30–35	41.8	14.3	6.42	−18.60	3.4	2
	O47	F	30–35	42.7	15.6	6.14	−19.16	3.1	2.5
	O48	F	25–30	44.0	16.0	6.81	−19.75	3.2	1.6
Sulmona S. Lucia	S1	F	45–50	41.3	16.5	5.61	−19.78	2.9	1.6
	S2	M	35–45	39.9	14.1	11.4	−20.69	3.3	1.8
	S3	F	45–50	34.9	17.3	7.54	−19.57	2.3	1.8
	S4	M	35–45	32.4	19.1	6.72	−19.30	2.0	1.8
	S5	F	25–35	40.9	16.0	6.30	−22.00	3.0	1.9
	S6	F	35–45	41.3	15.7	8.81	−18.69	3.1	2.2
	S7	M	18–25	42.7	16.7	10.88	−18.05	3.0	2.1
	S8	M	45–50	33.9	15.9	7.62	−20.11	2.5	1.6
	S9	M	18–25	39.8	15.8	7.95	−19.24	2.9	1.4
	S10	M	35–45	30.0	14.1	9.01	−17.72	2.5	1.2
	S11	F	18–25	26.2	11.6	7.45	−19.72	2.6	1.1
	S12	M	35–45	33.9	16.9	6.44	−21.96	2.3	1.2
	S13	F	45–50	44.0	16.7	6.58	−17.76	3.1	1.4
	S14	M	45–50	43.9	17.0	11.54	−17.74	3.0	1.1
	S15	F	18–25	42.2	16.4	11.95	−17.17	3.0	1.7
	S16	M	22–26	43.7	16.5	5.66	−19.71	3.1	2.1
	S17	M	25–35	39.5	14.8	7.09	−20.85	3.1	1.1
	S18	F	>50	40.0	15.3	8.57	−18.17	3.0	1.2
	S19	F	>60	38.7	14.1	8.15	−20.60	3.2	1.2
	S20	F	AD ^b^	—	—	—	—	—	0.9
	S21	F	AD ^b^	38.4	14.0	8.09	−20.69	3.2	1.9
	S22	F	AD ^b^	37.2	13.5	8.86	−17.54	3.2	1.8
	S23	F	36–44	43.0	15.9	7.95	−19.58	3.1	1.6
	S24	M	>50	42.6	15.9	8.46	−19.66	3.1	1.7
	S25	F	20	—	—	—	—	—	—
	S26	M	>50	42.6	15.4	7.89	−18.14	3.2	1.4
	S27	M	25–35	41.9	16.5	5.69	−19.78	3.0	1.4
	S28	M	>50	41.0	15.3	5.70	−19.76	3.1	1.1
	S29	M	>50	—	—	—	—	—	—
	S30	M	AD ^b^	—	—	—	—	—	—
	S31	M	>50	31.4	12.5	8.53	−19.01	2.9	1.1
	S32	F	AD ^b^	40.8	17.1	9.82	−17.76	2.7	1.1
	S33	M	AD ^b^	41.5	16.5	9.37	−19.60	2.9	1.2
	S34	F	>50	40.9	15.7	8.70	−19.24	3.0	1.1
	S35	M	AD ^b^	—	—	—	—	—	—
	S36	M	AD ^b^	—	—	—	—	—	—

Abbreviations: M, male; F, female. ^a^ No data collected. ^b^ Adult individual with indeterminate age.

**Table 3:** Isotope values (‰) of faunal samples from Gabii, La Sassa cave and Loreto Aprutino.

Animal [Reference]	δ^15^N (‰)	δ^13^C (‰)
*Capra aegagrus* [11]	5.2	−19.10
*Ovis aries* [11]	7.1	−21.30
*Ovis aries* [17]	5.7	−20.30
*Sus scrofa* [12]	6.7	−18.70
*Sus scrofa* [12]	4.8	−21.10
*Sus scrofa* [12]	6.1	−20.80

**Table 4:** Descriptive statistics for δ^13^C and δ^15^N values and Student’s *t*-test and Mann–Whitney *U*-test results for evaluating differences between male and female individuals in the samples from Opi Val Fondillo and Sulmona S. Lucia.

			Males			Females					
Sample	Stable Isotope		*n*	Mean	SD	*n*	Mean	SD	*t*	*U*	*p*
Opi Val Fondillo	Cluster #1										
		δ^13^C (‰)	6	−19.587	0.680	4	−19.513	0.046	—	3.500	0.068
		δ^15^N (‰)	6	12.603	0.274	4	12.548	0.165	—	10.000	0.670
	Cluster #2										
		δ^13^C (‰)	17	−18.617	0.493	15	−18.886	0.469	1.578	—	0.125
		δ^15^N (‰)	17	6.998	0.544	15	6.458	0.434	3.072	—	0.004
	Pooled clusters										
		δ^13^C (‰)	23	−18.870	0.606	19	−19.018	0.491	0.859	—	0.396
		δ^15^N (‰)	23	8.460	2.563	19	7.740	2.580	—	130.500	0.026
Sulmona S. Lucia		δ^13^C (‰)	16	−19.406	1.248	15	−19.093	1.365	−0.667	—	0.510
		δ^15^N (‰)	16	8.326	2.084	15	8.151	1.512	0.266	—	0.792

Abbreviations: n, number of individuals; Mean, overall measurement mean; SD, standard deviation; *t*, Student’s *t*-test; *U*, Mann–Whitney *U*-test; *p*, *p*-value.

**Table 5:** Descriptive statistics for δ^13^C and δ^15^N values and Kruskal–Wallis *H*-test results for evaluating differences between age groups in the samples from Opi Val Fondillo and Sulmona S. Lucia.

			Age Group (Years)										
			20–35			35–50			>50				
Sample	Stable Isotope		*n*	Mean	SD	*n*	Mean	SD	*n*	Mean	SD	*H*	*p*
Opi Val Fondillo	Cluster #1												
		δ^13^C (‰)	5	−19.560	0.079	4	−19.553	0.076	1	−19.560	—	0.112	0.946
		δ^15^N (‰)	5	12.668	0.242	4	12.455	0.202	1	12.650	—	1.669	0.434
	Cluster #2												
		δ^13^C (‰)	17	−18.802	0.549	10	−18.555	0.475	5	−18.916	0.187	2.144	0.342
		δ^15^N (‰)	17	6.777	0.669	10	6.705	0.417	5	6.716	0.481	0.018	0.991
	Pooled clusters												
		δ^13^C (‰)	22	−18.975	0.580	14	−18.840	0.646	6	−19.023	0.312	0.523	0.678
		δ^15^N (‰)	22	8.116	2.596	14	8.348	2.720	6	7.705	2.461	0.119	0.942
Sulmona S. Lucia		δ^13^C (‰)	9	−19.407	1.480	11	−19.355	1.330	7	−18.898	1.510	0.254	0.881
		δ^15^N (‰)	9	7.872	2.197	11	8.115	1.951	7	9.035	0.742	1.579	0.454

Abbreviations: n, number of individuals; Mean, overall measurement mean; SD, standard deviation; *H*, Kruskal–Wallis *H*-test; *p*, *p*-value.

**Table 6:** Mann–Whitney U-test results for evaluating differences of mean values of δ^13^C and δ^15^N between the samples from Opi Val Fondillo and Sulmona S. Lucia.

	Mean Rank			
Stable Isotope	Opi Val Fondillo	Sulmona S. Lucia	*U*	*p*
δ^13^C (‰)	40.18	32.69	517.500	0.136
δ^15^N (‰)	34.49	40.40	545.500	0.239

Abbreviations: *U*, Mann–Whitney *U*-test; *p*, *p*-value.

**Table 7:** Descriptive statistics for δ^13^C and δ^15^N values and Mann–Whitney *U*-test results for evaluating differences between Opi Val Fondillo and Sulmona S. Lucia with Loreto Aprutino, Gabii, and La Sassa cave sites.

		Comparison Test					
		Loreto Aprutino		Gabii		La Sassa	
Sample	Stable Isotope	*U*	*p*	*U*	*p*	*U*	*p*
Opi Val Fondillo vs.	δ^13^C (‰)	23.000	0.000	5.000	0.000	100.500	0.000
	δ^15^N (‰)	200.000	0.018	60.000	0.040	414.000	0.000
Sulmona S. Lucia vs.	δ^13^C (‰)	83.500	0.000	47.500	0.061	294.000	0.000
	δ^15^N (‰)	204.000	0.323	33.000	0.013	419.000	0.028

Abbreviations: *U*, Mann–Whitney *U*-test; *p*, *p*-value.

**Table 8:** Median of isotope values (‰) of human samples.

Necropolis	Italic Populations	δ^13^C (‰)	δ^15^N (‰)
Opi Val Fondillo	Samnites	−18.937	8.134
Sulmona S. Lucia	Peligni	−19.255	8.241
Loreto Aprutino	Vestini	−20.843	8.418
Gabii	Latini	−19.164	8.621
La Sassa	Latini	−19.832	9.434

## Data Availability

The database used and/or analyzed during the current study is available from the corresponding author upon reasonable request.
